# Transcriptional analysis of ESAT-6 cluster 3 in *Mycobacterium smegmatis*

**DOI:** 10.1186/1471-2180-9-48

**Published:** 2009-03-04

**Authors:** Anna Maciąg, Aurora Piazza, Giovanna Riccardi, Anna Milano

**Affiliations:** 1 Department of Genetics and Microbiology, University of Pavia, via Ferrata 1, 27100 Pavia, Italy; 2 Department of Biomolecular Sciences and Biotechnology, University of Milan, Via Celoria 26, 20133 Milan, Italy

## Abstract

**Background:**

The ESAT-6 (early secreted antigenic target, 6 kDa) family collects small mycobacterial proteins secreted by *Mycobacterium tuberculosis*, particularly in the early phase of growth. There are 23 ESAT-6 family members in *M. tuberculosis *H37Rv. In a previous work, we identified the Zur- dependent regulation of five proteins of the ESAT-6/CFP-10 family (*esxG*, *esxH*, *esxQ*, *esxR*, and *esxS*). *esxG *and *esxH *are part of ESAT-6 cluster 3, whose expression was already known to be induced by iron starvation.

**Results:**

In this research, we performed EMSA experiments and transcriptional analysis of ESAT-6 cluster 3 in *Mycobacterium smegmatis *(*msmeg0615*-*msmeg0625*) and *M. tuberculosis*. In contrast to what we had observed in *M. tuberculosis*, we found that in *M. smegmatis *ESAT-6 cluster 3 responds only to iron and not to zinc. In both organisms we identified an internal promoter, a finding which suggests the presence of two transcriptional units and, by consequence, a differential expression of cluster 3 genes. We compared the expression of *msmeg0615 *and *msmeg0620 *in different growth and stress conditions by means of relative quantitative PCR. The expression of *msmeg0615 *and *msmeg0620 *genes was essentially similar; they appeared to be repressed in most of the tested conditions, with the exception of acid stress (pH 4.2) where *msmeg0615 *was about 4-fold induced, while *msmeg0620 *was repressed. Analysis revealed that in acid stress conditions *M. tuberculosis rv0282 *gene was 3-fold induced too, while *rv0287 *induction was almost insignificant.

**Conclusion:**

In contrast with what has been reported for *M. tuberculosis*, our results suggest that in *M. smegmatis *only IdeR-dependent regulation is retained, while zinc has no effect on gene expression. The role of cluster 3 in *M. tuberculosis *virulence is still to be defined; however, iron- and zinc-dependent expression strongly suggests that cluster 3 is highly expressed in the infective process, and that the cluster contributes to the antigenic profile during the course of infection. Moreover, cluster 3 induction in acid stress conditions strengthens the hypothesis that cluster 3 is expressed in the course of infection.

In *M. smegmatis*, the expression of *msmeg0615 *and *msmeg0620 *genes is broadly similar in differing growth phases and in stress conditions, with the exception of acid stress (pH 4.2). Differences in expression between cluster 3 genes can be explained by the presence of internal promoters, both in *M. smegmatis *and *M. tuberculosis*.

## Background

The ESAT-6 (early secreted antigenic target, 6 kDa) family collects small mycobacterial proteins secreted by *Mycobacterium tuberculosis*, particularly in the early phase of growth. They were found in culture supernatant in the form of heterodimer with the related CFP-10 (culture filtrate protein, 10 kDa) proteins [1]. There are 23 ESAT-6 family members in *M. tuberculosis *H37Rv; located in 11 genomic *loci*, their genes have been named as *esxA-W *[2,3].

Inspection of the genetic neighbourhood revealed that in five out of eleven cases the *esx *genes are flanked by blocks of conserved genes. Besides *esx *genes, the other conserved regions encode PE and PPE proteins, ATP-dependent chaperones of the AAA family, membrane-bound ATPases, transmembrane proteins and serine proteases, which are known as mycosins [4]. These five ESAT-6 gene clusters were named regions 1 (*rv3866-rv3883c*), 2 (*rv3884c-rv3895c*), 3 (*rv0282-rv0292*), 4 (*rv3444c-rv3450c*) and 5 (*rv1782-rv1798*) [4].

The genomes of *M. tuberculosis *H37Rv, *M. bovis *and *M. bovis *BCG have been compared, and various regions of difference (RD) have been identified. One of these regions, designated as RD1, is a 9500 bp region that is absent in all *M. bovis *BCG strains [5]. This deletion entirely removes the genomic fragment from *rv3872 *to *rv3879c*. Among the lost genes are *esxB *(*rv3874*) and *esxA *(*rv3875*), which respectively encode CFP-10 and ESAT-6 proteins. This deletion is thought to be responsible for the primary attenuation of *M. bovis *to *M. bovis *BCG [5]. Moreover, using differential display to compare gene expression in *M. tuberculosis *H37Rv and H37Ra strains, Rindi *et al*. [6] showed that TB10.4 (the ESAT-6 protein coded by *rv0288*) is produced in the virulent, but not in the avirulent strain, a finding which suggests that this protein may be involved in functions that contribute significantly to the virulence of *M. tuberculosis*.

The secretion of CFP-10 and ESAT-6 proteins is promoted by a secretory apparatus that is encoded by the surrounding genes in the RD1 locus; these genes encode at least one transmembrane protein (Rv3877) and two AAA-family ATPases (Rv3870 and Rv3871) [7].

It is well known that CFP-10 and ESAT-6 are potent T-cell antigens that are recognized by TB patient sera [8], but their precise role in infection and virulence is still to be clearly defined. They are thought to possess a cytolytic activity and to be involved in cell-to-cell spread in the host, thus facilitating the dissemination of infection among macrophage and dendritic cells [9,10].

More recently, ESAT-6, CFP-10 and their complex were demonstrated to modulate the macrophage signalling pathway, and in particular the ERK 1/2 MAP kinase pathway [11]. The modulation was exerted by a strong inhibitory effect on the phosphorylation and subsequent activation of extracellular signal-regulated kinases 1/2 (ERK1/2) in the nucleus; this inhibition was achieved by an increase in phosphatase activity in the nucleus, which in turn caused dephosphorylation of pERK1/2 coming from the cytoplasm. The limitation of ERK 1/2 activation affected the expression of c-Myc, a key factor in macrophage activation, and thus downregulated the expression of LPS-inducible gene *c-myc*. Moreover, the ESAT-6/CFP-10 complex was shown to be able to inhibit the production of reactive oxidative species (ROS) and to interfere with LPS-induced ROS production. As a consequence, the downregulation of LPS-induced nuclear factor-kB (NF-kB) DNA binding activity [12] caused a reduced expression of several proinflammatory cytokines, such as TNF-α, IL-2, interferon-γ and nitric oxide synthase 2 [13,14].

The multiple duplicates of the ESAT-6 gene cluster found in the genome of *M. tuberculosis *H37Rv are also observable in the genomes of other mycobacteria, such as *M. bovis, M. leprae*, *M. avium*, and the avirulent strain *M. smegmatis*; it follows that the presence of the ESAT-6 gene cluster is a feature of some high-G+C Gram-positive bacteria [4]. In particular, the *M. smegmatis *genome contains three of the five ESAT-6 gene cluster regions, namely regions 1, 3 and 4, which in term of protein show 60 and 75% similarity to *M. tuberculosis *H37Rv [4]. No deletion, frameshifts or stop codons were identified in any of these genes, and it is therefore assumed that these regions are functional [4].

Besides, in *M. leprae *genome, which is believed to contain the minimal gene set required for pathogenesis, functional copies of both *ML0050/ML0049 *and *ML2532/ML2531 *(corresponding respectively to cluster 1 and 3 *esx *genes) are retained, a fact which suggests the importance of these proteins in mycobacterial virulence [4,15].

In a previous work, we identified thirty-two genes, which we hypothesised as being organized in 16 operons, under Zur (zinc uptake regulator) transcriptional control in *M. tuberculosis*; of these, five proteins belong to the ESAT-6/CFP-10 family (*esxG*, *esxH*, *esxQ*, *esxR*, and *esxS*) [16]. While *esxG *(CFP-10) and *esxH *(ESAT-6) are part of ESAT-6 cluster 3, *esxQ*, *esxR*, and *esxS *are physically associated, but do not belong to any of the five gene clusters [4]. Interestingly, the same gene cluster 3 is induced by iron starvation and is repressed by iron and IdeR [17]. Consistently with the notion that this gene cluster is dually regulated by Zur and by IdeR, we identified two different promoters upstream of its first gene (*rv0282*); one overlaps the Zur binding site, while the other overlaps the IdeR binding site [17].

In this research we performed EMSA experiments and transcriptional analysis of ESAT-6 cluster 3 in *M. smegmatis*. In contrast with what we had observed in *M. tuberculosis*, we found that in *M. smegmatis *ESAT-6 cluster 3 responds only to iron and not to zinc.

## Results

### Genetic organization of ESAT-6 cluster 3 and EMSA experiments on *msmeg0615* and *rv0282* promoters

**Figure 1:**
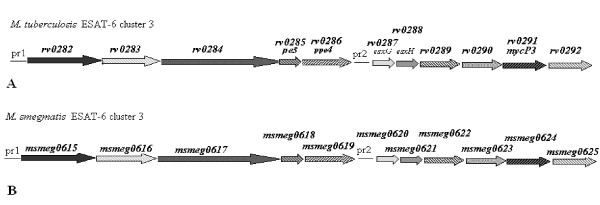
**Genetic organization of ESAT-6 cluster 3 in *M. tuberculosis *(A) and *M. smegmatis *(B)**. The position of the pr1 and pr2 promoters are indicated. The distance between *rv0286 *and *rv0287*, and between *msmeg0619 *and *smeg0620 *is arbitrary.

The transcriptional regulation of ESAT-6 cluster 3 (*rv0282-rv0292*) in *M. tuberculosis *is well documented [16,17]. The promoter region upstream of the *rv0282 *gene (pr1) was found to be regulated by Zur protein in a zinc-dependent manner, as well as by IdeR in an iron-dependent manner [16,17]. *M. smegmatis *ESAT-6 cluster 3 presents a similar genetic organization, and comprises 11 genes numbered *msmeg0615*-*msmeg0625 *(Figure 1) (Genome sequence with accession number CP000480).

**Figure 2:**
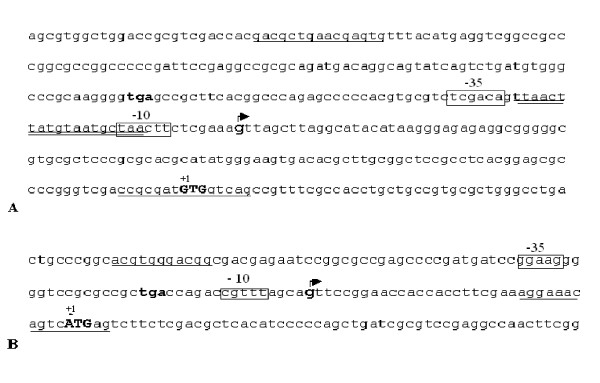
**Promoter regions and transcriptional start sites of *M. smegmatis *ESAT-6 cluster 3**. Sequences upstream of the *msmeg0615 *(A) and *msmeg0620 *(B) genes: primer sequences utilized for the cloning of promoter regions are underlined; stop codons of the upstream gene are in bold; translational start codons (+1) are in bold capital letters; transcriptional start sites are in bold and indicated with an arrow; hypothetical -35 and -10 regions are boxed; IdeR binding site is double underlined.

Sequence analysis of the *msmeg0615 *upstream region revealed the presence of a hypothetical IdeR binding region (5'-TTAACTTATGTAATGCTAA-3') (double underlined in Figure 2A), while no evident region of homology with *M. tuberculosis *Zur DNA binding box (5'-TATTGAAAATCATTTTCATTA-3') could be found.

To define metal-dependent regulation of cluster 3, we cloned *M. smegmatis zur *(*msmeg4487*) and *ideR *(*msmeg2750*) genes into the pGEX-6P-1 vector. The corresponding proteins were expressed in *Escherichia coli *XL1-Blue and purified by on-column digestion with PreScission Protease (GE Healthcare). The quality of purified proteins was checked on SDS polyacrylamide gel (12–15%) and the molecular sizes were confirmed. Purified *M. smegmatis *Zur protein showed the molecular weight of 14 kDa, similarly to *M. tuberculosis *Zur, while IdeR protein showed the molecular weight of 25 kDa (data not shown).

**Figure 3:**
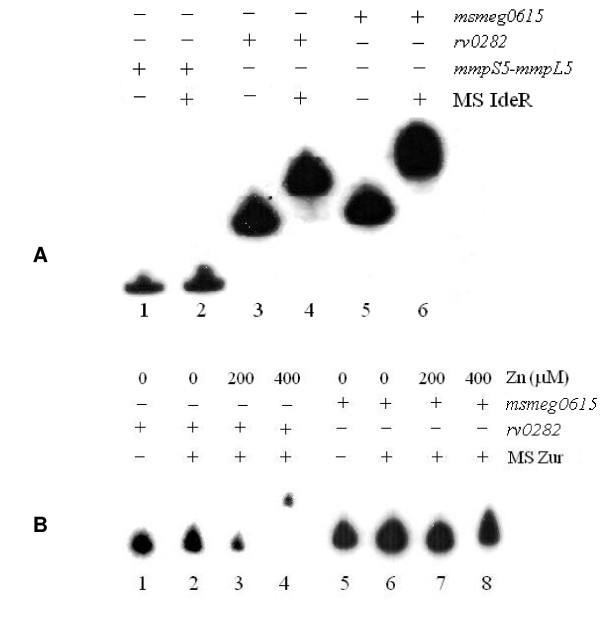
**EMSA experiments on *M. smegmatis *and *M. tuberculosis *pr1 promoter with *M. smegmatis *IdeR (A) and Zur (B) proteins**. (A) Migration of different DNA fragments representing the upstream region of the following genes: *mmpS5-mmpL5 *(unrelated fragment) (lanes 1–2), *rv0282 *(lanes 3–4), *msmeg0615 *(lanes 5–6), in the absence (-) and in the presence (+) of *M. smegmatis *IdeR. (B) EMSA experiments on the promoter region of *M. tuberculosis rv0282 *(lanes 1–4) and *msmeg0615 *(lanes 5–8) with *M. smegmatis *Zur. Lanes 1 and 5, negative control (without protein); lanes 2 and 6 no metal; lanes 3 and 7 200 μM Zn; lanes 4 and 8 400 μM Zn.

In order to verify the regulation of *msmeg0615-msmeg0625 *cluster, we used the *M. smegmatis *purified proteins in EMSA experiments on the *rv0282 *and *msmeg0615 *upstream regions (Figures 3A, B). As shown in Figure 3A, *M. smegmatis *IdeR was able to bind both promoter regions, while *M. smegmatis *Zur seemed to recognize and efficiently retard only the *rv0282 *promoter, but not the corresponding region of *M. smegmatis *(Figure 3B). The data suggest that cluster gene regulation differs between *M. tuberculosis *and *M. smegmatis*; we particularly note the lack of zinc regulation for the *msmeg0615 *promoter.

### Determination of the transcriptional start site and effects of different metal ions on pr1

5' RACE experiment was performed to further characterize the *M. smegmatis msmeg0615 *(pr1) promoter region. Similarly to *M. tuberculosis *[11], the hypothetical start site, mapping at -114 upstream of the *msmeg0615 *gene (indicated with the arrow in Figure 2A), identified a *consensus *promoter sequence that partially overlapped the palindromic sequence (5'-TTAACTTATGTAATGCTAA-3') (Figure 2A), which was highly homologous to the previously identified *M. tuberculosis *IdeR binding site [16,17].

**Figure 4:**
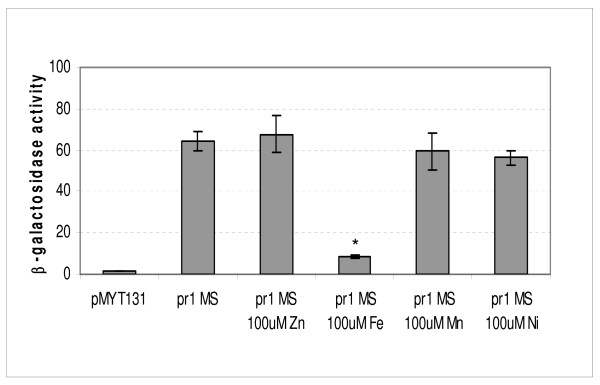
***msmeg0615 *(pr1) promoter activity**. β-galactosidase activity of cultures grown in Sauton medium in the presence of varying divalent metal ions. The values, expressed as nanomoles of *o-nitrophenol-β*-D-galactopyranoside converted to *o*-nitrophenol min^-1 ^mg^-1 ^of protein, represent the average and the standard deviation of three independent clones. * indicates that values are significantly different from the control value (p < 0.01).

β-galactosidase assays were performed to better define the activity of the *msmeg0615 *promoter (pr1). A fragment extending from -292 to +8, which was obtained by amplification with Pr1MSF and Pr1MSR primers (primer sequences are underlined in Figure 2A), and which contained the promoter region, was cloned in fusion with the *lacZ *gene into the integrative plasmid pMYT131. β-galactosidase activity was tested in Sauton medium, in the presence and in the absence of metal ions. In accordance with EMSA results, those data clearly demonstrated that *M. smegmatis *cluster 3 is repressed by iron, while other metal ions like zinc, nickel and manganese have no effect on its expression (Figure 4).

### 5'-RACE and transcriptional analysis of pr2

Cluster 3 gene organization seems to exclude the presence of internal promoter regions with one exception; the distance between the *ppe *(*rv0286, msmeg0619*) and *esxG *(*rv0287*, *msmeg0620*) coding regions suggested the presence of an internal putative promoter upstream of *M. tuberculosis esxG *and the corresponding homologous *msmeg0620 *gene (Figures 1, 2B). The short *rv0287-rv0288 *and *msmeg0620-msmeg0621 *intergenic regions were not analyzed, as the two genes had previously been reported to be cotranscribed [18]. To determine whether the putative pr2 promoter was present, we amplified the *rv0286-rv0287 *and the *msmeg0619*-*msmeg0620 *intergenic regions (Figure 2B) and cloned them into pMYT131. The recombinant plasmids were transformed into *M. smegmatis*, and β-galactosidase activity was measured.

**Figure 5:**
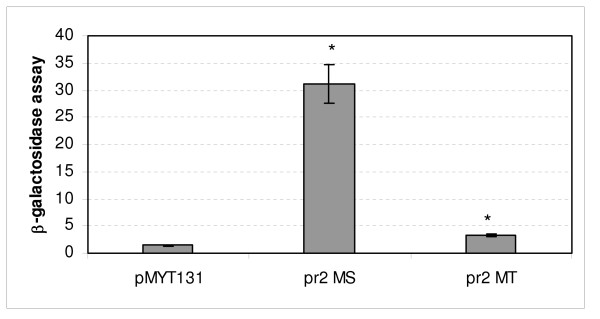
***msmeg0620 *(pr2 MS) and *rv0287 *(pr2 MT) promoter activity**. β-galactosidase activity of *msmeg0620 *and *rv0287 *(pr2) in *M. smegmatis *cultures grown in 7H9 medium at mid-log phase. The value represents the average and the standard deviation of three independent clones. * indicates that values are significantly different from the control value (p < 0.01).

As shown in Figure 5, the data suggest the presence of an alternative promoter just upstream of the *esx *genes, as enzymatic activity, particularly for the *msmeg0619*-*msmeg0620 *intergenic region was significantly higher than that measured in the control culture (*M. smegmatis *transformed with the empty vector). The data regarding *M. tuberculosis *are less clear, since detectable promoter activity was low.

To better define promoter sequences, we performed 5' RACE experiment. The transcriptional start site, indicated with an arrow in Figure 2B, mapped at -34 upstream of the *msmeg0620 *translational start codon. Although no SigA promoter *consensus *sequence was observed in the upstream region, we could found hypothetical -10 and -35 sequences that resembled those reported as to be possibly recognizable by *M. tuberculosis *SigH factor [19]. We did not identify any pr2 promoter sequence in *M. tuberculosis*, as the 5' RACE experiments were unsuccessful.

### Quantitative PCR on *msmeg0615 *and *msmeg0620 *genes and their homologs in *M. tuberculosis*

*M. smegmatis *mc^2^155 was grown at different growth phases and in different stress conditions; RNA was extracted, retrotranscribed and used in relative quantitative PCR (qPCR) experiments. To determine the effect of pr1 and pr2 activity on cluster 3 genes, we analysed the expression of two representative coding regions, *msmeg0615 *and *msmeg0620*, located immediately downstream of these promoters. *sigA *(*mysA, msmeg2758*) gene, which codes the primary sigma factor, was used as a normalizing reference. The normalized values were referred to gene level expression of *M. smegmatis *as grown in 7H9 medium to mid-log phase (OD_600 _= 0.8).

**Figure 6:**
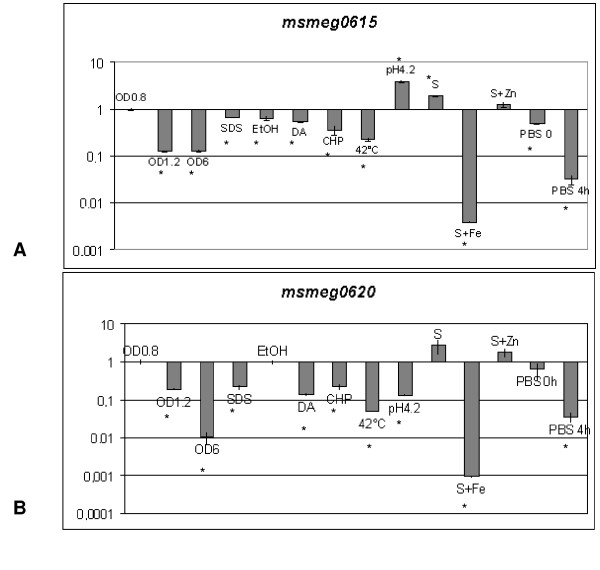
**Expression of *msmeg0615 *and *msmeg0620 *genes**. Level of expression of *msmeg0615 *(A) and *msmeg0620 *(B) genes in differing growth and stress conditions relative to the expression of the same gene in 7H9 culture in mid-log phase (OD = 0.8) (taken as 1). The level of *sigA *transcript was used to normalize the amount of RNA. The value represents the average and the standard deviation of three independent reactions. * indicates that values are significantly different from the control value (p < 0.01).

The data reveal (Figures 6A, B) that the expression of *msmeg0615 *and *msmeg0620 *is essentially similar in most of the conditions analysed. The results confirm that metal deficiency (Sauton medium, previously treated with Chelex 100) is associated with ESAT-6 cluster 3 derepression; the presence of zinc (S+Zn) has no effect on gene expression, while iron clearly determines gene repression (S+Fe).

Both genes appear to be repressed in most of the other conditions, such as late phase of growth (OD_600 _= 6), nutrient starvation (PBS0 and PBS4), surface stress (SDS), ethanol stress (EtOH), oxidative stress (DA and CHP), and heat shock (42°C). Curiously, the *msmeg0615 *and *msmeg0620 *genes respond differently to acid stress (pH 4.2), with the former induced by about 4-fold, and the latter appearing to be repressed.

*rv0282 *and *rv0287 *gene expression was monitored by means of qPCR to verify pH-dependent regulation in *M. tuberculosis*. With the *sigA *gene as a normalizing reference, the data revealed a higher level of expression in acid stress conditions than was the case for 7H9 standard medium with respective inductions of about 3-fold (2.97 ± 0.08) for *rv0282 *and 1.5-fold (1.48 ± 0.2) for *rv0287*.

β-galactosidase activity in *M. smegmatis *cultures, transformed with pMYT131 derivatives carrying *M. smegmatis *and *M. tuberculosis *pr2 regions, revealed that promoter activities were significantly (about two-fold) lower under acid stress than in control conditions (data not shown).

## Discussion

ESAT-6 (early secreted antigenic target, 6 kDa) proteins, including the previously mentioned CFP-10 (10 kDa short-term culture filtrate protein), form a large family that is defined on the following base: basis of protein size (about 100 amino acids); the occurrence of the cognate genes in pairs; their location downstream of a *pe *and *ppe *gene pair, which are coding mycobacterial protein with a characteristic proline-glutamic (PE) and proline-proline-glutamic (PPE) motif.

The interest in ESAT genes derives from the observation that *esxA *and *esxB *genes are comprised in the RD1 (region of difference 1), whose deletion is thought to be responsible for the primary attenuation of *M. bovis *in *M. bovis *BCG [5]. Complementation experiments have demonstrated that mutations that abolish production or secretion of RD1 ESAT-6 proteins confer an attenuated phenotype in various animal models, which in turn suggests that ESAT-6/CFP-10 play an important role in survival and multiplication of *M. tuberculosis *within the host cell [20,21].

Moreover, ESAT-6 proteins have been identified as strong targets for human B- and T-cell response, a finding which stimulates great interest in the potential of these antigens for vaccine use [22]. Besides EsxA and EsxB, EsxH (Rv0288), included in cluster 3, has also been identified as a strong antigen in TB patient and BCG vaccinated donor [23]. Two other ESAT proteins (Rv3017c, or EsxQ and Rv3019c, or EsxR), despite their high degree of identity with Rv0288, display a unique epitope pattern [24]. These observations strengthen the hypothesis that these genes could encode proteins whose functions are similar, but whose recognition by the immune system differs; differential expression of individual genes could lead to antigenic variation, which would help mycobacteria to escape from the host defence. To better understand *esx *genes function it is important to investigate their expression in varying conditions and in differing phases of the infective process.

*esx *genes were also identified in other mycobacteria; in particular the fast growing *M. smegmatis *contains three ESAT-6 gene clusters, which correspond to the previously identified regions 1 (encompassing region between *msmeg0057 *and *msmeg0083 *genes), 3 (*msmeg0615*-*msmeg0625*) and 4 (*msmeg1534-msmeg1538*) of *M. tuberculosis*. The finding that bacteria carrying ESAT-6 genes live in varying environmental niches suggests that, besides virulence, these proteins could have a more general role in mycobacterial physiology.

To better define the putative role of cluster 3 in mycobacterial pathogenicity and physiology, we decided to study ESAT cluster 3 gene regulation in *M. smegmatis *and in *M. tuberculosis*. As the *rv0282 *promoter region had been previously characterized [16], we analysed *msmeg0615 *promoter region activity. Our results suggest that regulation differs in these organisms; while in *M. tuberculosis *gene cluster 3 is controlled by IdeR and Zur regulators in an iron- and zinc-dependent manner, in *M. smegmatis *only IdeR-dependent regulation is retained, while zinc has no effect on gene expression. Iron is a growth limiting factor both in the environment and during human infection. In mammalian hosts this metal is bound to high affinity iron-binding proteins, and abnormal high iron levels in serum are associated with exacerbation of the disease [25]. It is worth noting that the differences in ESAT-6 cluster expression 3 in *M. tuberculosis *and *M. smegmatis *could be due to differences in the life styles of these organisms. As a pulmonary pathogen, *M. tuberculosis *has to confront with a zinc-deficient environment, as this metal's concentration is low in lung alveoli [26]. While ESAT-6 cluster 1 is known to be essential to virulence, the role of cluster 3 is still to be defined; nevertheless, iron- and zinc-dependent expression strongly suggest a high level expression in the lung during the infective process, and hence a contribution to the antigenic profile throughout the course of infection [22].

To better understand the expression of ESAT-6 cluster 3 genes, it was important to verify whether internal promoters appear within this region; in both organisms, the presence of promoter upstream of *msmeg0620 *and *rv0287 *coding regions suggests that gene expression within ESAT-6 gene cluster could be differential. To better define the effect of each promoter on overall *esx *gene regulation, we compared *msmeg0615 *and *msmeg0620 *expression in varying conditions by means of relative quantitative PCR. As an internal control to normalize loaded RNA we used *sigA*, which encodes the mycobacterial major sigma factor [27,19]. *sigA *is widely used as a standard in qPCR because its expression is constitutive in various growth phases and under differing stress conditions. An approximate 3-fold decrease in *sigA *transcript was reported in *M. tuberculosis *during the stationary growth phase [28]; these data do not seem to affect our results significantly, as we observed increased repression of this promoter in the stationary phase.

The expression of *msmeg0615 *and *msmeg0620 *genes is essentially similar; they appear to be repressed in most of the tested conditions, with the exception of acid stress (pH 4.2). These data suggest the presence of two transcriptional units: the first, regulated by pr1 (*msmeg0615 *promoter), encompasses the whole cluster, while the second, regulated by pr2, includes the *msmeg0620 *downstream genes. Although previous studies [16] noted the coordination of all genes expression within cluster 3 under Zur regulation, divergence between *rv0282 *and *rv0287 *induction levels under acid stress and the appearance of an internal promoter also suggest that two overlapping transcriptional units exist.

As regards the hypothetical role of the CFP-10/ESAT-6 complex in escaping from the phagosomal compartment of professional phagocytic cells [29,30], the finding of cluster 3 gene induction in acidic pH condition is surely noteworthy. Acidification may indeed be a signal for the induction of genes needed in phagosome survival.

A previous transcriptional analysis by means of microarray failed in the identification of *rv0282 *and *rv0287 *among *M. tuberculosis *genes induced under acid stress [31]. This discordance could be explained with different sensitivity of the methodologies used in these investigations.

Both IdeR and iron-regulated genes were previously reported to be upregulated during macrophage infection [32,33]. This apparent contradiction can be explained by direct or indirect inhibition exerted by environmental acid on IdeR function. Indeed, to date no data suggest the presence of an alternative pH-dependent promoter upstream of ESAT-6 cluster 3; *msmeg0615 *and *rv0282 *gene induction could be indirect, presumably as an effect of the environment on IdeR function or stability. Differential gene expression inside the ESAT-6 cluster could be related to the presence of the internal promoter pr2, whose activity diminishes under acid stress. As pr2 seems to be a weak promoter, its effect in *M. tuberculosis *could be less evident, while in *M. smegmatis *it could effectively reduce pr2-regulated genes expression. Unfortunately, it was not possible to identify pr2 promoter sequence in *M. tuberculosis*, as 5' RACE experiments were unsuccessful; the probable reason is low expression levels. In *M. smegmatis*, no SigA *consensus *sequence could be found upstream of the 5' end of the transcript. We can hypothesize the involvement of an alternative sigma factor; indeed, this region showed sequence (boxed in Figure 2B) that resembled the sequence putatively recognized by *M. tuberculosis *SigH [19,34]. However, in this organism, SigH is induced by heat shock and oxidative stress [34] and we are accordingly unclear as to the meaning of this observation. On the other hand, a bioinformatics search has predicted the existence of 26 sigma factors in *M. smegmatis*, with a significant enrichment in the SigH subfamily [35]. These paralogous members might have acquired specific functions, and might be induced in varying as yet unidentified conditions.

## Conclusion

Our data suggest that ESAT-6 cluster 3 regulation in mycobacteria varies. Particularly, in *M. tuberculosis *the gene cluster is induced by iron and zinc starvation and is repressed by IdeR and Zur regulators. In *M. smegmatis*, only IdeR-dependent regulation is retained, while zinc has no effect on gene expression. Differences in expression could be due to diversity in the life styles of these organisms. Iron is a limiting growth factor in the environment and during human infection, but as a pulmonary pathogen *M. tuberculosis *also contend with a zinc-deficient environment. Although the role of cluster 3 is not defined, induction in iron- and zinc-deficient condition, as pertain in the lung, strongly suggests a high level expression of this cluster during the infective process.

Both in *M. tuberculosis *and in *M. smegmatis *we identified an internal promoter just upstream of the *esx *genes (respectively *rv0287 *and *msmeg0620*). These promoters seem to be repressed under acid stress, and thus to contribute to differential expression of this gene cluster in varying environmental conditions.

## Methods

### Strains, media and growth conditions

*Escherichia coli *XL1-Blue was grown in Luria Bertani (LB) medium [36] at 37°C. When required, antibiotics were added at the following concentrations: ampicillin, 100 μg/ml; streptomycin, 50 μg/ml, tetracycline, 12.5 μg/ml.

*M. smegmatis *mc^2^155 was grown in liquid Middlebrook 7H9 supplemented with ADN (2% glucose, 5% BSA, 0,85% NaCl) and 0,05% Tween 80 or solid Middlebrook 7H11 medium (Difco) supplemented with Middlebrook oleic acid-albumin-dextrose-catalase (OADC) (BBL) at 37°C.

For studies of promoter regulation as mediated by metals, *M. smegmatis *strains were grown in Sauton medium treated with Chelex 100 resin (Sigma-Aldrich), as previously described [37]. After Chelex 100 treatment and sterilization, Sauton medium was integrated with 1 mM MgSO_4 _and, in some cases, with other metals, as indicated in Results.

When required, streptomycin was added at the concentration of 10 μg/ml.

### Expression and purification of recombinant *M. smegmatis *Zur and IdeR proteins

*M. smegmatis zur (furB) *and *ideR *genes were amplified by PCR with the respective primers RG329-RG330 and IdeR F- IdeR R (Table 1), and cloned into pGEX-6P-1 vector. *E. coli *XL1-Blue cultures, carrying the recombinant plasmid containing the *ideR *gene, were grown to log phase (OD_600 _= 0.5–0.8), induced by addition of 0.1 mM IPTG and incubated at 37°C for 3 hours. *M. smegmatis *Zur protein was induced by addition of 0.1 mM IPTG and incubated overnight at 26°C. Cells were subsequently harvested by centrifugation, washed with 1× PBS (8 g/l NaCl, 0.2 g/l KCl, 1.44 g/l Na_2_HPO_4_, 0.24 g/l KH_2_PO_4_) and stored at -20°C.

**Table 1:** Primer sequences

Primer	Sequence	Purpose
IdeR F IdeR R	5'TTGGATCCATGAACGATCTTGTCGATAC-3' 5'-CGGAATTCTCAGACCTTCTCGACCTTG-3'	cloning of *ideR *coding region into pGEX-6P-1

RG329 RG330	5'-CCGGGATCCATGACGGGCGCGGT-3' 5'-CCGGAATTCTCACGTCTGGTTCCCG-3'	cloning of *zur *coding region into pGEX-6P-1

Rv0282-1 Rv0282-2	5'-CGGGATCCCGCAACACCCTGGTC-3' 5'-CGGGTACCCGCTGTCTCCTTCACC-3'	EMSA on *rv0282 *promoter region

mmp3 mmp7	5'-GCACGCTTGAGAGTTCC-3' 5'-TGCCACTTTCGGGTC-3'	EMSA on *mmpS5 *promoter region

Pr1MS F Pr1MS R	5'-CCAGTACTGACGCTGGAACGAGTG-3' 5'-CCAAGCTTCTGACCACATCGCGG-3'	EMSA and cloning of *msmeg0615 *promoter region into pMYT131

Pr2MS F Pr2MS R	5'-CCAGTACTACGCTGACCGGCGAC-3' 5'-CCAAGCTTCTCATGACTGTTTCCTTTC-3'	Cloning of *msmeg0620 *promoter region into pMYT131

Pr2MT F Pr2MT R	5'-CCAGTACTCAACGAGCCCGAGGCG-3' 5'-CCAAGCTTCTCATAACATCTCTCC-3'	Cloning of *rv0287*(*esxG*) promoter region into pMYT131

RA1 RA2	5'-GACCACGCGTATCGATGTCGAC(T)_16_V-3' 5'-GACCACGCGTATCGATGTCGAC-3'	5' RACE PCR reactions

Ms0615-RT MS0615-1 Ms0615-2	5'-GTCGACGACGGCCGGGGTG-3' 5'-CCGATCCACGCGTCGCAC-3' 5'-GTCGTGTGCGAGATGGGTC-3'	5' RACE for *msmeg0615*

Ms0620-RT Ms0620-1 Ms0620-2	5'-GTCGAGCAGCGCATTGAC-3' 5'-CGAGACCTCGACGAAACG-3' 5'-GCATGCGCGGCCTGGAAG-3'	5' RACE for *msmeg0620*

Ms0615 A Ms0615 B	5'-GGCCTGACGGTCAACG-3' 5'-ATCCACGCGTCGCACT-3'	qPCR for *msmeg0615*

Ms0620 E Ms0620 F	5'-CAGGCCGCGATGAGTT-3' 5'-TCGAGCAGCGCATTGA-3'	qPCR for *msmeg0620*

mysA F mysA R	5'-CGTCGCCGATGGTCTG-3' 5'-CCACGCCCGAAGAGC-3'	qPCR for *M. smegmatis sigA (mysA)*

Rv0282 C Rv0282 D	5'-AGGTGTCGCGGCTGAA-3' 5'-GGTCCCGCAAACACCA-3'	qPCR for *Rv0282*

Rv0287 A Rv0287 B	5'-CTGATGGCGGCACACGA-3' 5'-CAGAAACCGGGCATGG-3'	qPCR for *Rv0287*

sigA F2 sigA R2	5'-CGCGAAAAACCATCTG-3' 5'-GATCAGCCCCAGGTTG-3'	qPCR for *tuberculosis sigA*

For protein purification, cell pellet from 250 ml of culture was resuspended in 4 ml of 1× PBS (Phosphate-buffered saline) and sonicated on ice. The lysate was centrifuged for 30 min at 12000 × *g *at 4°C and the supernatant mixed with 0.5 ml of Glutathione Sepharose 4B resin (GE Healthcare), previously equilibrated with ten volumes of the same buffer.

The resin was then packed on column by gravity and the unbound fraction was recovered. The column was washed extensively with PBS monitoring proteins elution spectrophotometrically; when the flow-through reached an OD_280 _near 0, digestion Buffer (50 mM Tris HCl pH 7.0, 150 mM NaCl) was applied to the column. After equilibration of the resin in this buffer, PreScission Protease (GE Healthcare) was added. After overnight digestion, the samples were collected and analyzed by SDS-PAGE to estimate the yield and purity of the proteins.

### EMSA experiments on ESAT-6 cluster 3 pr1 of *M. smegmatis*

*M. smegmatis *Zur and IdeR proteins were used in EMSA experiments on the *msmeg0615 *promoter region, obtained by PCR with Pr1MSF and Pr1MSR as primers.

The corresponding region of *M. tuberculosis rv0282*, amplified with Rv0282-1 and Rv0282-2 primers, was used as a positive control for Zur regulation [16]. As a negative control, we used the promoter region of unrelated genes (*mmpS5-mmpL5*), obtained by amplification with mmp3 and mmp7 primers. *mmpS5-mmpL5 *were previously reported as IdeR-independent iron-repressed genes [17].

DNA fragments were labelled with [**γ**^32^P] dATP by means of T4 Polynucleotide Kinase (Promega) and used as probes. Subsequently, 20 μl of binding reaction mixture containing 150 ng (6 pmol) of IdeR protein and 20 fmol of labelled probe (20 mM Tris-HCl pH 8.0, 50 mM KCl, 2 mM DTT, 5 mM MgCl_2_, 50 μg/ml bovine serum albumin, 50 μg/ml salmon sperm DNA, 10% glycerol, 200 μM NiSO_4_), was incubated for 30 min at room temperature. EMSA experiments with *M. smegmatis *Zur protein were performed in the same way as for *M. tuberculosis *Zur [16]. Reaction mixtures were loaded onto a nondenaturing 6% polyacrylamide gel containing 1× TA [36]. Gels were run at 140 V at room temperature, dried, and exposed to Hyperfilm (GE Healthcare).

### 5' RACE

For 5' rapid amplification of cDNA ends (5' RACE), 1 μg of *M. smegmatis *RNA and 20 pmol of specific primer (Ms0615-RT or Ms0620-RT) (reported in Table 1), were incubated at 70°C for 5 min, chilled on ice, and then reverse transcribed with ImProm-II Reverse Transcriptase (Promega) in accordance with the manufacturer's instructions. Finally, the reactions were purified with Wizard SV Gel and PCR Clean-up System (Promega) and incubated at 37°C for 30 min in the presence of 2 mM dATP and 20 U of Terminal Deoxynucleotidyl Transferase (Promega) to add a poly(A) tail to the 3' end. The product of the reaction was used as a template in the first PCR reaction performed with RA1 and Ms0615-1 or Ms0620-1 primers. The amplification products were then used as templates for seminested PCRs, with RA2 and an internal oligonucleotide primers (Ms0615-2 or Ms0620-2, respectively). The PCR fragments were purified with Wizard SV Gel and PCR Clean-up System (Promega) and sequenced by BMR Genomics (www.bmr-genomics.it).

### Promoter identification

Region upstream of the *msmeg0615*, *msmeg020 *and *rv0287 *(*esxG*) genes were amplified with specific primers, as reported in Table 1. Each fragment was purified with Wizard SV Gel and PCR Clean-up System (Promega), digested with *ScaI *and *HindIII *and ligated into the integrative vector pMYT131 (kindly provided by D. Ghisotti). pMYT131 is a pSM128 derivative, obtained by partial digestion with *HindIII *and relegation, which removes the first 14 *lacZ *codons. Mycobacterial promoter regions, including gene start codons, were cloned in translational fusion with the reporter gene *lacZ*. β-galactosidase activity was measured on cellular extracts, as previously described [38].

### Analysis of mRNA by qRT-PCR

*M. tuberculosis *RNA (kindly provided by R. Provvedi), was extracted from cultures under stress condition, as indicated below.

Two independent *M. smegmatis *mc^2^155 cultures at mid log-phase (OD_600 _= 0.8) were used for expression analysis under stress conditions. Aliquots of 5 ml were treated for 90 min at 37°C as follows: 0.1% sodium dodecyl sulphate (SDS) (detergent stress), 5 mM diamide (DA) (oxidative stress), 1 mM cumene hydroperoxide (CHP) (oxidative stress), 2.5% ethanol (EtOH). Acid stress was examined by washing of the culture, resuspension of the same in complete 7H9 medium at pH 4.2 (previously acidified with HCl), and incubation for 90 min at 37°C. For heat shock, the aliquot was incubated for 90 min at 42°C.

For nutrient starvation conditions, aliquots were washed twice with PBS (Phosphate-buffered saline) and resuspended in the same buffer. One aliquot was immediately recovered (PBS 0), while the other was incubated at 37°C for 4 h. For metal-dependent expression, *M. smegmatis *mc^2^155 was grown in Sauton medium, as previously described [35]. Overnight cultures were grown in Sauton medium previously treated with Chelex 100 (Sigma- Aldrich) in conditions of metal deficiency or of iron or zinc ion supplementation with at the final concentration of 100 μM. Aliquots of *M. smegmatis *grown in 7H9 medium were collected at varying OD_600_values and used for expression analysis at differing growth phases.

RNA was isolated by means of Rneasy Mini Kit (Qiagen). After DNAse treatment, all samples were tested by conventional PCR to rule out DNA contamination.

1 μg of total *M. tuberculosis *or *M. smegmatis *RNA and 0.5 μg of random primers were heated for five minutes at 70°C, chilled on ice and then reverse-transcribed with ImProm-II Reverse Transcriptase (Promega), in accordance with the manufacturer's instructions. Samples corresponding to 25 ng of RNA were used in each PCR reaction in a final volume of 20 μl. Each reaction was performed in triplicate. Negative controls were included.

Experiments were performed with cDNA derived from two independent cultures per treatment.

Quantitative PCR were performed by means of QuantiTect SYBR Green PCR Master Mix (Qiagen) on a Rotor Gene 6000 (Corbett Life Science). The specificity of the reactions was checked by analysis of the melting curve. *M. tuberculosis *and *M. smegmatis sigA *gene was used as an internal invariant control for the normalization of change in gene expression.

Expression data were calculated with the -2^ΔΔCt ^method (ΔC_t _= C_t sample _- C_t control_) and were reported as -fold change in gene expression of each sample normalized to the invariant gene (*sigA*) relative to the untreated (culture in mid-log phase) control.

### Statistical analysis

Where appropriate, statistical analysis was performed by Student's *t *test, and significance is indicated in the text.

## Authors' contributions

AMa performed protein purifications. EMSA experiments, promoter cloning and enzymatic assays. AP performed transcriptional analysis. GR performed experimental coordination and helped in the draft of the manuscript. AMi performed transcriptional analysis, participated in the design of the study and drafted the manuscript. All authors read and approved the final manuscript.
